# In Vitro Evaluation of Apical Transportation Induced by Single‐ and Dual‐Thermally Treated NiTi Instruments

**DOI:** 10.1155/ijod/6168069

**Published:** 2026-02-04

**Authors:** Francesco Puleio, Rosario Pirri, Jose Aranguren, Vincenzo Tosco

**Affiliations:** ^1^ Dipartimento di Scienze Biomediche Odontoiatriche e delle Immagini Morfologiche e Funzionali, Università degli Studi di Messina, Messina, SIcily, Italy, unime.it; ^2^ Independent Researcher, Messina, Sicily, Italy; ^3^ Campus de Alcorcon, Community of Madrid, Universidad Rey Juan Carlos, Alcorcon, Spain, urjc.es; ^4^ Department of Life Sciences, Link Campus University, Health and Health Professions, Rome, Italy

**Keywords:** endodontic, endodontic instrument, martensitic instrument, thermal treated alloy

## Abstract

**Introduction:**

Martensitic endodontic instruments, thanks to their flexibility, produce less apical transportation compared to austenitic instruments. Recently, a sequence of instruments featuring a dual thermal treatment applied separately to the tip and the shank was introduced with the aim of achieving a tip with enhanced cutting capacity and a more flexible body. The increased stiffness at the tip could potentially lead to greater apical transportation. The aim of this study was to compare apical transportation produced by SlimShaper (single thermal treatment) and SlimShaper PRO (dual thermal treatment) nickel–titanium (NiTi) instruments (Zarc4Endo, Spain) in standardized simulated canals.

**Methods:**

Forty‐four standardized J‐shaped resin blocks were used, divided into two groups (*n* = 22): Group A (shaped with SlimShaper up to size 25/0.04) and Group B (shaped with SlimShaper PRO up to size 25/0.04). Each block was photographed before and after shaping, and the images were superimposed to evaluate apical transportation. Data normality was verified with the Shapiro–Wilk test, and homogeneity of variances was assessed with Levene’s test. Since assumptions were met, comparisons between the two groups were performed using the independent Student’s *t* test, with the level of significance set at *p*  < 0.05.

**Results:**

Mean apical transportation was 0.08423 mm (SD 0.01049) for SlimShaper and 0.08609 mm (SD 0.01102) for SlimShaper PRO, with no statistically significant difference between groups (*p* = 0.57).

**Conclusions:**

Under the conditions of this study, the dual‐thermally treated SlimShaper PRO instruments did not produce greater apical transportation than the single‐thermally treated SlimShaper instruments.

## 1. Introduction

A complete and centered root canal preparation, performed while respecting the anatomical complexity of the endodontic system, is essential for achieving predictable clinical success [1]. Appropriate shaping enhances irrigant penetration and efficacy, supporting the biological objectives of root canal therapy [2]. The advent of nickel–titanium (NiTi) rotary instrumentation represented a major milestone, markedly reducing procedural errors and allowing safer and more efficient preparation of curved canals [3]. This improved adaptability enables clinicians to maintain working length and preserve residual dentin while enhancing the centering ability of canal preparation [4, 5].

Since their introduction, NiTi instruments have undergone constant refinement through various surface and thermomechanical processes, including electropolishing, electric discharge machining, and thermal treatments [6]. Thermal modification enhances key mechanical properties such as flexibility and resistance to cyclic fatigue [7]. These treatments alter the proportion of martensitic and austenitic phases, often imparting characteristic colors (e.g., blue, gold) associated with an increased martensitic content and improved mechanical behavior [8, 9].

Recently, a sequence of NiTi instruments with three distinct thermal treatments (SlimShaper; Zarc4Endo, Spain) was introduced, designed to provide a progressive increase in martensitic content across the sequence. As an evolution of this system, the SlimShaper PRO was developed featuring a dual thermal profile, in which the tip and the shaft undergo different heat treatments (dual‐wire design). According to the manufacturer, the shaft contains a higher martensitic phase than the tip, potentially influencing flexibility and mechanical behavior during shaping. However, these characteristics are manufacturer‐reported and were not evaluated in the present study.

Apical transportation—defined as the displacement of the physiological canal terminus, typically toward the outer aspect of canal curvature—remains a critical complication associated with rotary instrumentation [10, 11]. Once transportation occurs, restoring the original anatomy becomes difficult or impossible, particularly in curved canals [12–14]. This deviation may compromise cleaning, weaken root structure, and predispose to complications such as zipping, apical perforation, or extrusion of debris and irrigants [15–17]. Therefore, evaluating the potential of new thermally treated instruments to maintain canal centering is essential for assessing their clinical safety.

Although the metallurgical characteristics of thermally treated NiTi instruments have been widely investigated, there is limited evidence comparing shaping behavior between instruments that share the same design but differ in their thermal profiles—particularly those with dual‐thermal treatment at distinct portions of the instrument. No published data currently assess whether this dual‐profile configuration may affect apical transportation.

The aim of the present study was therefore to compare apical transportation produced by SlimShaper (single‐thermal treatment) and SlimShaper PRO (dual‐thermal treatment) using a standardized simulated canal model and pre/postoperative image superimposition. The null hypothesis was that there would be no significant difference in apical transportation between the two instrument systems.

## 2. Materials and Methods

### 2.1. Sample Collection and Preparation

Twenty‐two Slimshaper and 22 Slimshaper PRO were observed through 10x magnification (Carl Zeiss OPMI Pro Ergo, Germany) in order to check for irregularities or production imperfections. 100% of the total was selected for the study, presenting no observable irregularities or imperfections. The minimum number of instruments to be included in this research was identified through a power analysis, considering the division into two groups. Sample size calculation was performed prior to the study using 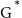
Power 3.1 (Heinrich Heine University, Düsseldorf, Germany). A two‐tailed independent *t*‐test was selected, with an alpha level of 0.05, a power of 0.90, and an effect size of 0.90 based on preliminary data from similar studies. The analysis indicated that a minimum of 22 samples per group was required. Accordingly, 44 resin blocks were included and randomly assigned to the two experimental groups. The SlimShaper and SlimShaper PRO instruments share identical design characteristics, including cross‐sectional geometry, number of threads, helix angle, taper, tip size, and core diameter. According to the manufacturer, the only difference between the two systems is the thermomechanical treatment: a single‐thermal profile for SlimShaper and a dual‐thermal profile applied independently to the tip and shaft of SlimShaper PRO. Forty‐four standard clear resin J‐shaped endoblocks (Endo‐Training‐Bloc, Dentsply Maillefer, Ballaigues, Switzerland) were used in this research and randomly assigned into Groups A and B. Randomization was performed using GraphPad Prism, version 10.1.2 (GraphPad Software, LLC, San Diego, CA, USA). Blocks were listed with a number, while instruments were assigned the letter A for Slimshaper and B for Slimshaper Pro. Another researcher placed the matching codes inside an opaque envelope, and samples were randomly extracted, as visual randomization is not possible due to the chromatic difference between the instruments.

Preoperative pictures of the endoblocks were taken using a Nikon D7500 (Nikon Corporation, Tokyo, Japan) with a macro lens (Nikkor Z MC 105 mm, Nikon Corporation, Tokyo, Japan) and a 15 MS‐1 Metz flash (Metz Werke GmbH & Co. KG, Zindorf, Bayern, Germany) and saved as JPEG files. The correct positioning of the camera was secured using a tripod, and a remote was used to take the pictures. The blocks were positioned in a plastic structure that did not allow axial rotation or translation of the samples.

The blocks were assessed for initial apical patency with a K‐file 10/0.02 (Dentsply Tulsa Dental, Tulsa), set 1 mm beyond the working length (16 mm). The blocks were shaped as follows:

• Group A (*n* = 22): the blocks were shaped with Slimshaper (ZS1, ZS2, and ZS3 at 500 rpm and 3 ncm torque)
• Group B(*n* = 22): the blocks were shaped with Slimshaper PRO (ZS1, ZS2, and ZS3 at 500 rpm and 3 ncm torque).

The canal was irrigated with 1 mL of alcohol using an endodontic syringe and a 30 G endodontic needle (Endo Star, Poldent, Warsaw, Poland).

After each instrument (ZS1, ZS2, and ZS3), the canal was irrigated with 2 mL of alcohol using a 30 G needle at the rate of 1 mL/10”. In addition, after each instrument, a K‐file 10/0.02 was inserted 1 mm beyond the working length.

After the shaping phase, the canals were rinsed with 3 mL of alcohol using a 30 G needle at the rate of 1 mL/10” and dried using paper points (Zarc4endo, Gijón, Asturias, Spain) and a gentle blast of air.

Pictures were taken positioning the shaped blocks on the structure. All the images were stored as JPEG files and elaborated using GIMP 2.10.38 (The GIMP Development Team, USA). The original root canal was highlighted with a blue filter on the preoperative image, and the postoperative image was faded by reducing its opacity to allow visual superimposition.

**Figure 1:**
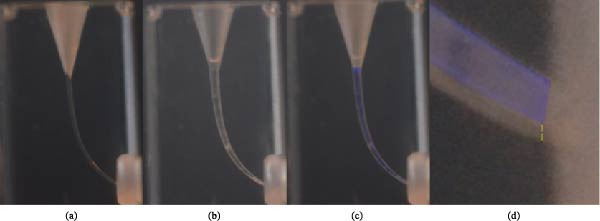
(a) Resin block used; (b) shaped resin block; (c) superposition of the preoperative (blue filter) and postoperative image; (d) measurement performed.

Measurements were carried out using ImageJ/Fiji (National Institute of Mental Health, Bethesda, Md, USA) software, calibrated with a millimeter ruler to allow scale conversion (pixels to millimeters). The measurement was taken at the apical portion of the canal, perpendicular to the axis of the apical portion and parallel to its terminal portion (Figure 1).

### 2.2. Statistical Analysis

Data were collected and tabulated for all samples. For each Groups (A and B), the mean values and standard deviations (SDs) were calculated. Data normality was verified using the Shapiro–Wilk test, and homogeneity of variances was assessed with Levene’s test. Since the assumptions were satisfied, comparisons between groups were performed using the independent Student’s *t*‐test. The level of statistical significance was set at *p*  < 0.05.

## 3. Results

The mean apical transportation for Group A was 0.084 mm (SD = 0.010), while for Group B it was 0.086 mm (SD = 0.011). The results of the individual measurements for each block are reported in Table 1.

**Table 1:** Results of measurements of apical transport at D0.

Block (A)	Apical transport^a^	Block (B)	Apical transport
1	0.063	3	0.076
2	0.080	6	0.089
4	0.075	7	0.070
5	0.091	8	0.092
10	0.097	9	0.095
11	0.083	13	0.084
12	0.088	14	0.080
16	0.100	15	0.101
18	0.081	17	0.075
19	0.069	20	0.090
21	0.092	22	0.086
24	0.079	23	0.082
25	0.084	28	0.094
26	0.074	29	0.079
27	0.095	30	0.105
32	0.085	31	0.073
35	0.106	33	0.108
38	0.072	34	0.087
40	0.078	36	0.081
42	0.091	37	0.065
43	0.088	39	0.092
44	0.082	41	0.090

^a^Values expressed in millimeters.

No statistically significant difference was found between the two groups (*p* = 0.57; 95% CI –0.009 to 0.005).

## 4. Discussion

Endodontics has greatly benefited from ongoing research into different NiTi alloys and their performance characteristics, improving efficiency and reducing operator fatigue [9, 12]. However, despite these advantages, the use of NiTi instruments is not without risks. Procedural complications such as ledges, apical transportation, and straightening of curved canals remain clinically relevant issues that can compromise treatment outcomes [18–22]. The curvature of the apical portion is a decisive factor influencing the likelihood of transportation; smaller radii and greater degrees of curvature increase the tendency of the instrument to straighten the canal [23, 24]. For this reason, standardized curved resin blocks were selected in the present study to reproduce challenging clinical conditions in a reproducible manner. The use of resin blocks for evaluating shaping outcomes is well documented in the literature [25, 26]. Their uniformity and transparency facilitate pre‐ and postoperative photographic superimposition, allowing for straightforward assessment of canal modification [27, 28]. In simulated canal studies, the choice of irrigant must consider material compatibility: sodium hypochlorite, chlorhexidine, and other clinical solutions can soften, alter, or discolor resin surfaces, leading to geometric distortion or loss of optical clarity during imaging. For this reason, alcohol is commonly employed in resin models, as it ensures adequate lubrication and debris removal while preserving the structural stability and translucency of the blocks. Its use, therefore, reflects a methodological necessity intrinsic to resin‐based experiments rather than an attempt to reproduce clinical irrigation conditions.

Most previous studies comparing apical transportation have analyzed instruments with different geometries, tapers, or kinematics, whereas relatively few investigations have evaluated instruments sharing the same design but differing in thermal treatment [29–31].This represents an important methodological strength of the present research: SlimShaper and SlimShaper PRO are identical in design, taper, and operational parameters, enabling isolation of thermal treatment as the sole variable under comparison. According to the manufacturer, the SlimShaper PRO sequence features a dual thermal profile—one applied to the shaft and another to the tip—resulting in different phase compositions along the instrument. These manufacturer‐reported characteristics suggest differences in the mechanical behavior of specific segments of the file, but the present study did not assess properties such as cutting efficiency, and no claims regarding enhanced performance can be drawn from our experimental data.

The results of this investigation showed mean apical transportation values of 0.08423 mm (SD = 0.01049) for the single‐thermally treated instruments (Group A) and 0.08609 mm (SD = 0.01102) for the dual‐thermally treated instruments (Group B), with no statistically significant difference between groups (*p* = 0.57). These values are consistent with previous studies that used similar standardized resin blocks and measurement approaches [32]. A possible explanation for the minimal differences observed is that the manufacturer‐reported modification of the tip’s thermal profile in the SlimShaper PRO may not be sufficient to alter its shaping behavior under the conditions of this model. Alternatively, the increased martensitic content reported in the shaft may compensate for any localized variation at the tip, maintaining the overall flexibility and centering ability of the instrument. From a metallurgical perspective, both instruments likely operated within mixed martensitic–austenitic phases at room and intracanal temperatures. NiTi alloys under these conditions typically show a balance between flexibility and stiffness that supports centering, and this shared phase behavior may have contributed to the similar transportation values recorded for both systems.

Another important consideration in the interpretation of these findings is the intrinsic hardness of the simulated canals. Resin blocks offer lower hardness and elastic modulus compared with natural dentine. As demonstrated by Khalilak et al., canal wall hardness significantly influences shaping outcomes, with higher‐hardness substrates generating greater resistance to instrument movement and potentially amplifying deviations [33, 34]. In the context of this study, the absence of differences between the two sequences in a low‐hardness material suggests that clinically relevant deviations are unlikely to be more pronounced in dentine, where higher structural stiffness would oppose uncontrolled file deviation more effectively. Although resin blocks may underestimate the absolute magnitude of transportation, the comparative findings between SlimShaper and SlimShaper PRO are likely to remain valid under clinical conditions, or potentially even more favorable.

From a clinical perspective, preserving canal anatomy and minimizing apical transportation is essential for maintaining apical patency, optimizing irrigant penetration, and reducing iatrogenic complications [27]. Given the similar shaping behavior observed between the two sequences in this standardized model, the dual‐thermal profile of the SlimShaper PRO—although not evaluated in terms of performance advantages—did not negatively influence transportation. Nonetheless, further research on extracted teeth or clinical scenarios is required to assess whether differences may emerge under variable temperature conditions, complex anatomies, or different mechanical stresses. Intracanal temperature fluctuations, variations in dentine hardness, operator‐dependent pressure, and dynamic loading may influence the response of thermomechanically treated instruments, potentially revealing subtle differences not detectable in resin models. Therefore, while the null hypothesis could not be rejected in this in vitro study, additional investigations using natural teeth and advanced imaging modalities remain necessary to fully understand the clinical implications of dual thermal treatment.

## 5. Limitations

This study presents several limitations. First, apical transportation was evaluated using two‐dimensional photographic superimposition, which, although widely used and reproducible, cannot fully capture the three‐dimensional complexity of canal geometry. More advanced imaging modalities such as micro‐computed tomography (micro‐CT) would provide volumetric information, improved spatial resolution, and a more comprehensive analysis of shaping outcomes. Second, resin blocks were used to standardize canal anatomy, but they differ from dentine in hardness, elastic modulus, and structural behavior. Their lower hardness may underestimate absolute transportation values; however, this characteristic also suggests that the absence of differences between groups in this material is unlikely to invert in harder substrates. Third, the irrigation protocol employed alcohol instead of sodium hypochlorite or chlorhexidine because clinical irrigants interact negatively with resin surfaces. While necessary in this model, the protocol does not replicate clinical irrigation and limits direct clinical translatability. Finally, although the sample size was calculated a priori, larger studies involving natural teeth and different canal anatomies are warranted to confirm these findings.

## 6. Conclusions

Within the limitations of this in vitro study, dual‐thermally treated SlimShaper PRO instruments did not produce greater apical transportation than single‐thermally treated SlimShaper instruments. The difference between the two sequences was not statistically significant, and the null hypothesis cannot be rejected. Further studies using natural teeth and three‐dimensional evaluation methods are needed to confirm these findings.

## Author Contributions

**Francesco Puleio**: conceptualization, methodology, formal analysis, writing – original draft, writing – review and editing, project administration; **Rosario Pirri**: methodology, investigation, data curation; **Jose Aranguren**: validation, writing – review and editing; **Vincenzo Tosco**: conceptualization, supervision, writing – review and editing.

## Funding

The authors received no external funding for this study.

## Conflicts of Interest

The authors declare no conflicts of interest.

## Data Availability

The data that support the findings of this study are available from the corresponding author upon reasonable request.
